# Highly-Sensitive In Vitro Bioassays for FSH, TSH, PTH, Kp, and OT in Addition to LH in Mouse Leydig Tumor Cell

**DOI:** 10.3390/ijms241512047

**Published:** 2023-07-27

**Authors:** Danièle Klett, Lucie Pellissier, Didier Lomet, Flavie Derouin-Tochon, Vincent Robert, Thi Mong Diep Nguyen, Anne Duittoz, Eric Reiter, Yann Locatelli, Joëlle Dupont, Hugues Dardente, Frédéric Jean-Alphonse, Yves Combarnous

**Affiliations:** INRAe, CNRS, UMR “Physiologie de la Reproduction & des Comportements”, Tours University, Inria, 37380 Nouzilly, France; daniele.klett@inrae.fr (D.K.); lucie.pellissier@inrae.fr (L.P.); didier.lomet@inrae.fr (D.L.); nguyenthimongdiep@qnu.edu.vn (T.M.D.N.); anne.duittoz@univ-tours.fr (A.D.); eric.reiter@inrae.fr (E.R.); yann.locatelli@mnhn.fr (Y.L.); joelle.dupont@inrae.fr (J.D.); hugues.dardente@inrae.fr (H.D.); frederic.jean-alphonse@inrae.fr (F.J.-A.)

**Keywords:** mouse Leydig Tumor Cell, g-protein coupled receptor, luteinizing hormone, follicle-stimulating hormone, thyroid-stimulating hormone, parathyroid hormone, kisspeptin, β2 adrenergic ligands, melatonin, oxytocin

## Abstract

We demonstrate here that highly sensitive in vitro bioassays for FSH, TSH, and PTH can be set up in mouse Leydig Tumor Cells (mLTC), in addition to the normal LH/CG bioassay, after they were transfected with expression vectors encoding the corresponding Gs Protein-Coupled Receptors (GsPCR), such as FSHR, TSHR, or PTHR. Although the β2 adrenergic receptor is also a GsPCR, its expression in mLTC led to a significant but very low cAMP response compared to those observed with FSH, TSH, or PTH. Similarly, after transfection of the GiPCR MT1 melatonin receptor, we did not observe any inhibitory effect by melatonin of the LH or hCG stimulation. Interestingly, after transfection of mLTC with the human kisspeptin receptor (hKpR), which is a GqPCR, we observed a dose-dependent synergy of 10^−12^–10^−7^ M kisspeptin variants with a fixed concentration of 0.3 nM LH or hCG. Without any exogenous receptor transfection, a 2 h preincubation with OT or AVP led to a dose-dependent cAMP response to a fixed dose of LH or hCG. Therefore, highly sensitive in vitro bioassays for various hormones and other GPCR ligands can be set up in mLTC to measure circulating concentrations in only 3–10 µL of blood or other body fluids. Nevertheless, the development of an LHRKO mLTC cell line will be mandatory to obtain strict specificity for these bioassays to eliminate potential cross-reaction with LH or CG.

## 1. Introduction

Many GPCR hormonal ligands are present at very low concentrations in the blood stream, from low picomolar to nanomolar ranges, hindering the detection of only their bioactive forms. We have previously set up a highly specific and sensitive in vitro bioassay for Luteinizing Hormones (LH) and Chorionic Gonadotropins (CG) from numerous mammalian species [1]. This assay makes use of a real-time intracellular luminescent cAMP assay in the mouse Leydig Tumor Cell line (mLTC) that was established by R.V. Rebois [2], confirming that these cells express functional endogenous LH/CG receptors.

In this LH/CG bioassay, we were able to accurately measure from 30 pM of LH/CG by preincubating the cells for 2 h with 10^−3^ M IBMX, 10^−5^ M forskolin (FSK), and 10^−8^ M oxytocin (OT), before introducing the reference hormones and samples to be tested for a 1 h incubation and real-time cyclic AMP luminescence measurement [1].

In the present work, we aimed to further develop this bioassay for OT or its paralog arginine vasopressin (AVP), which both interact with the endogenously expressed OT receptor (OTR) during the preincubation period before stimulation with a fixed concentration of LH. We also explored whether hormones, neuropeptides and ligands other than LHs, CGs, OT and AVP (all having endogenous receptors in mLTC),can be monitored at low concentrations, using the same highly sensitive assay, by expressing exogenous receptors in these cells.

With this objective in mind for various Gs-protein coupled receptor (GsPCR) ligands, we transfected the mLTC cells with expression vectors encoding exogenous GsPCR to develop new, highly sensitive in vitro bioassays for Follicle-Stimulating Hormone (FSH), Thyroid-Stimulating Hormone (TSH) and Parathyroid Hormone (PTH), respectively. Moreover, we also transfected another GsPCR, the β2 adrenergic receptor, and two non-GsPCRs, i.e., the melatonin receptor MTNR1A, and kisspeptin receptor KISS1R, acting through GiPCR and GqPCR, respectively, for an attempt to set up bioassays for the corresponding hormones, melatonin (MLT) and kisspeptin (Kp), respectively.

Finally, to measure the low concentrations of each aforementioned hormone in extremely small volumes of biological fluids, it was important to determine the dose-dependent curve for the reference preparations in plasma, serum or other biological fluid of interest, at dilutions similar to those used for the determination of unknown samples.

## 2. Results

### 2.1. LH Bioassays

As previously reported, highly sensitive and specific assays of LHs from different mammalian species, but not from birds or fishes, have been set up in mLTC [1]. As these assays are aimed in particular at measuring LH concentrations in blood serums or plasmas, we studied the cAMP luminescence responses to a range of ovine LH concentrations in 1/10 diluted serums or plasmas from various species.

**Figure 1:**
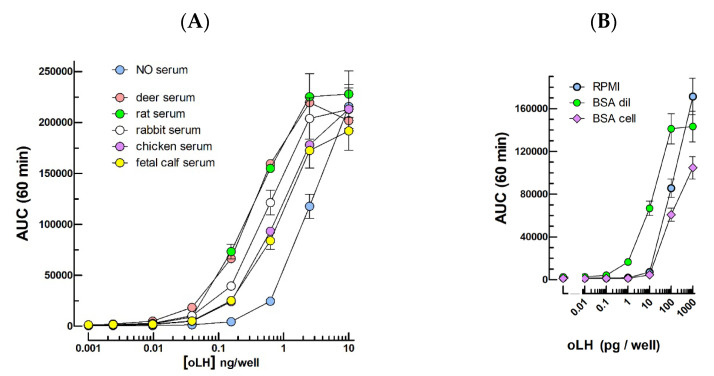
Effect of 1/10 diluted sera from various species or BSA on the cAMP response to ovine LH. (**A**) Effect of sera. Using the standard protocol including forskolin and oxytocin [1] in the mLTC medium, the sera from various species were present at 1% final concentration. (**B**) Effect of BSA (0 or 1 mg/mL) during oLH dilution (BSA dil) compared to the same final BSA quantity added separately to the cell medium (BSA cell). AUC: Area Under 60 min kinetics Curves.

Figure 1A shows that the ovine LH (oLH) dose–responses curves are displaced towards lower concentrations when the hormone is diluted in 1/10 serums (i.e., 1% final concentration in the culture medium). The serums from all the species tested did so, including that from chicken, even if we observed that chicken LH (ckLH) did not cross-react in this assay. BSA (1 mg/mL) during the serial dilution of oLH had the same detection limit lowering effect as the sera (Figure 1B). When the same quantity of BSA is added to the cells, separately from oLH, it does not exhibit such an effect but, instead, shows a slight lowering of the maximum response amplitude.

### 2.2. FSH Bioassays

**Figure 2:**
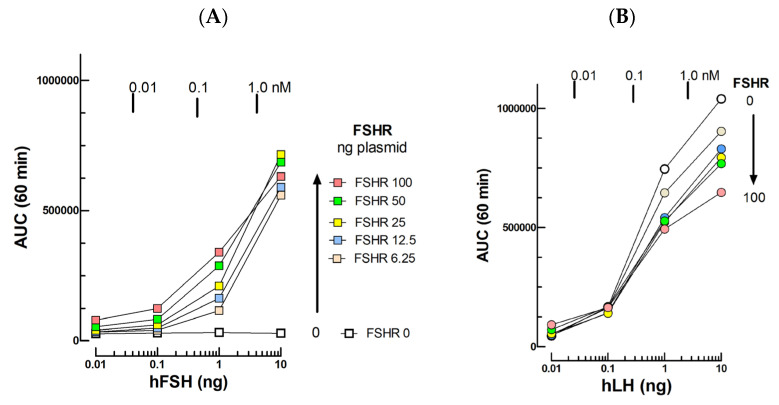

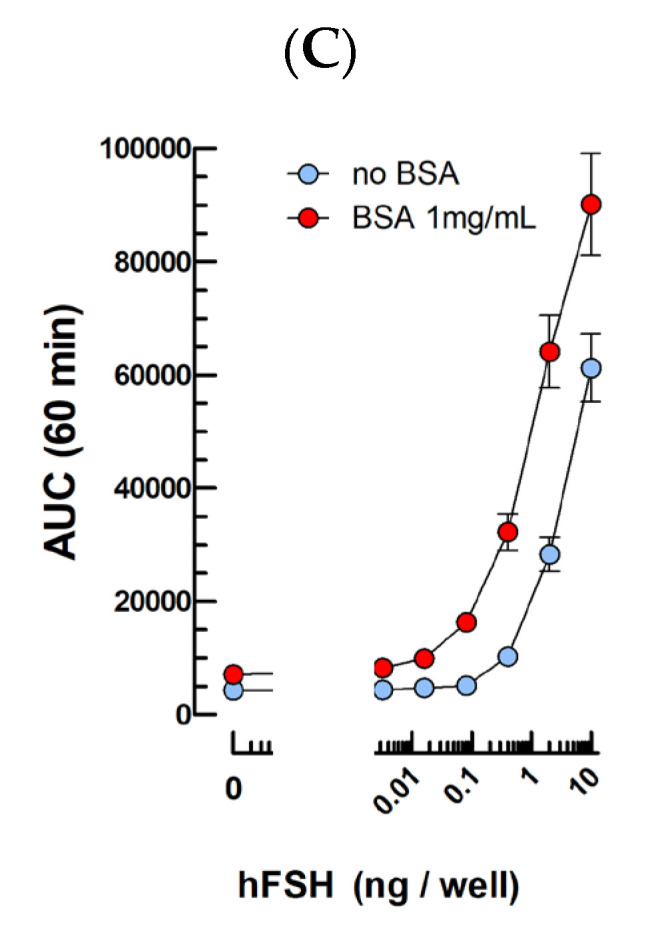
Upper panel: Cyclic AMP response to hFSH (**A**) and hLH (**B**) after transfection of mLTC with increasing concentrations of hFSHR expression plasmid. The mLTC cells were transfected with no or 6.25 to 100 ng hFSHR plasmid before challenging with 10–1000 pM concentrations hFSH (**A**) or hLH (**B**). (**C**) hFSH dose–response curves when the hormone is diluted in RPMI medium alone (blue dots) or completed with 1 mg/mL BSA (red dots).

Following transfection of an expression vector for the human FSH receptor (hFSHR) at various concentrations, we observed a plasmid dose-dependent cAMP response to 10^−12^–10^−9^ M hFSH (Figure 2A). The Kd of hFSH binding to hFSHR transfected in HEK293 cells has been previously shown to be 1.37 nM [3]. The limit of hFSH detection around 0.1 nM in the present assay is in strong agreement with this Kd value.

The cells were still found to respond to 10^−12^–10^−9^ M hLH but the response amplitude diminished as a function of the hFSH plasmid concentration (Figure 2B). The genetic deletion of LHR in a stable LHRKO mLTC cell line will be mandatory to set up a specific FSH assay.

It is interesting that the mLTC cells become sensitive to FSH following this protocol but this is accompanied by a ~50% decrease in the cells’ response amplitude to LH. As for oLH, the presence of BSA (1 mg/mL) during the preparation of serial dilutions of hFSH had a significant lowering effect on its detection limit in this assay (Figure 2C).

### 2.3. TSH Bioassays

**Figure 3:**
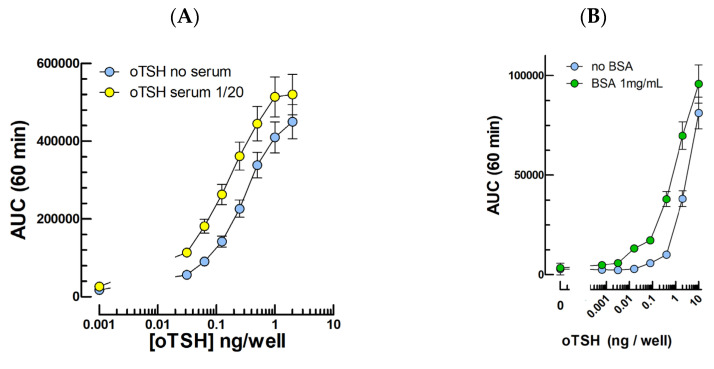

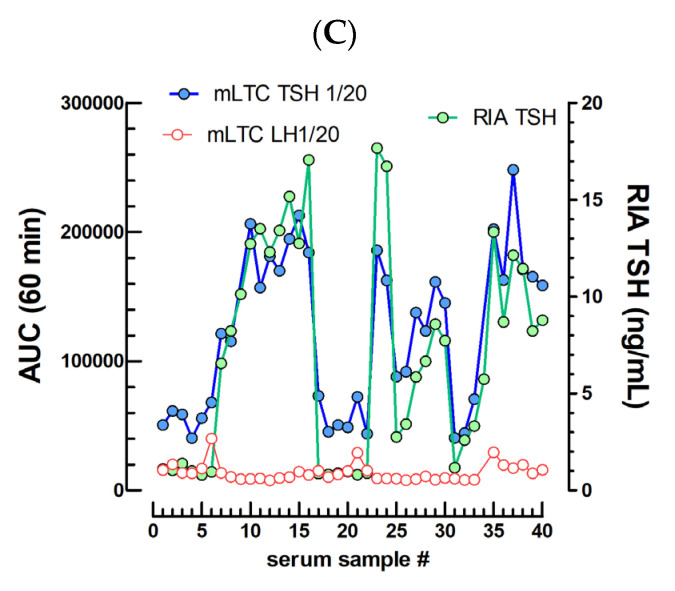
Cyclic AMP response to ovine TSH in mLTC transfected with the oTSHR. (**A**): oTSH dose–response curves in the absence or presence of 1/20 ovine serum (0.5% final concentration). (**B**): oTSH dose–response curves when the hormone is diluted in RPMI medium alone (blue dots) or completed with 1 mg/mL BSA (green dots). (**C**): oTSH concentration profiles in serums at 20-fold dilution measured by the in vitro bioassay compared to oTSH RIA. The red curve shows the AUC in the absence of TSHR (thus due only to oLH in the samples).

Following transfection of an expression vector for the ovine TSH receptor (oTSHR) at various concentrations, we selected the minimal plasmid concentration providing the optimal response to ovine TSH. In this condition, we observed dose-dependent cAMP responses to 10^−12^–10^−9^ M oTSH in the absence or presence of 1/20 ovine serum (Figure 3A) or 1 mg/mL BSA (Figure 3B) during TSH serial dilution.

As for oLH and hFSH, the presence of BSA (1 mg/mL) during the preparation of serial dilutions of oTSH had a significant lowering effect on its detection limit in this assay (Figure 3B).

### 2.4. PTH Bioassays

**Figure 4:**
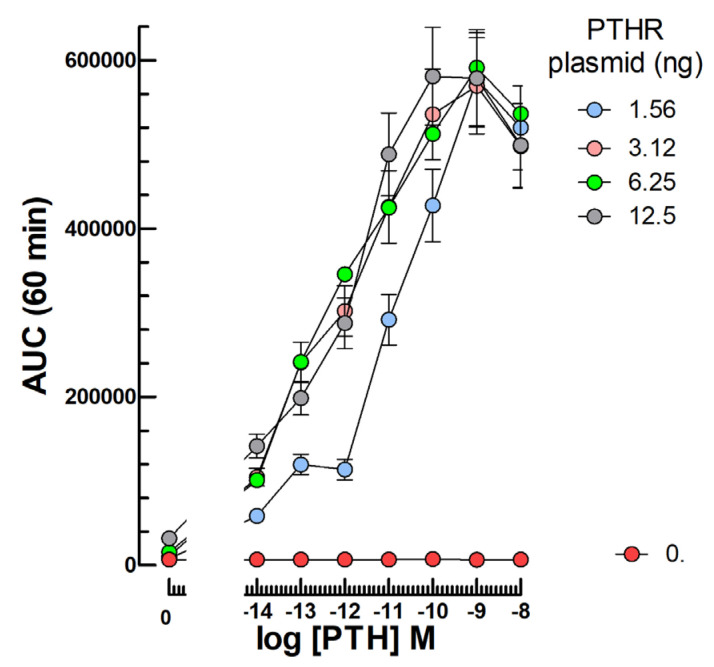
Dose-dependent cyclic AMP response to 10^−14^–10^−8^ M hPTH in mLTC transfected with hPTHR. The mLTC cells were transfected with 0 or 1.56 to 12.5 ng PTHR plasmid per well before challenging with 10^−14^–10^−8^ M hPTH 1–34.

Following transfection of an expression vector for the human PTH receptor (hPTHR) at various concentrations, we observed a plasmid dose-dependent cAMP response to 10^−14^–10^−8^ M PTH 1–34 (Figure 4).

At and above 3.12 ng transfected PTHR plasmid per well, an optimal response to PTH with an EC50 around 10^−12^ M was observed (Figure 4). Until now, we have not yet tested human blood serums or plasmas in this PTH assay but its sensitivity appears compatible with published normal circulating PTH concentrations in humans (14–65 pg/mL, i.e., ~1–6 pM) [4,5].

### 2.5. Catecholamine Bioassays

**Figure 5:**
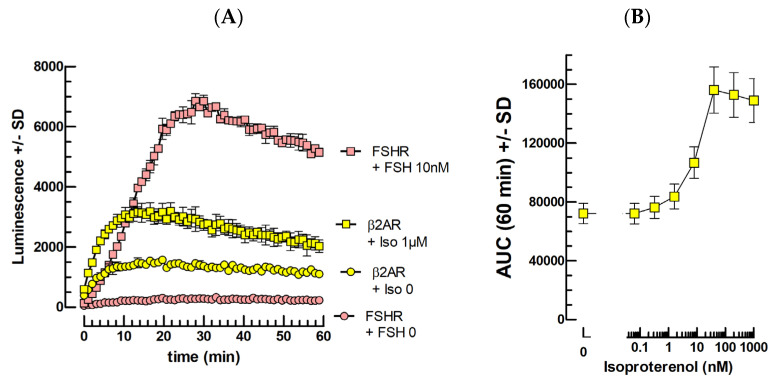
Kinetics of ISO/β2AR and FSH/FSHR luminescence responses (**A**) and Isoproterenol dose–response (**B**).

Following transfection of an expression vector for the human β2 adrenergic receptor (hβ2AR) at 100 ng/well, we observed a weak plasmid dose-dependent cAMP response to 10^−12^–10^−7^ M isoproterenol (Figure 5) compared to that elicited by FSH in similar conditions (Figure 5A). Additionally, a weak but significant cAMP response in the absence of ISO was observed, suggesting an intrinsic activity of the hβ2AR when expressed in mLTC cells in these conditions.

Figure 5A shows that there is a low but significant intrinsic activity of the hβ2AR receptor transfected in mLTCs. For comparison, in the same experiment, the transfection of a similar quantity of FSHR plasmid did not lead to any intrinsic activity of FSHR in this assay. Moreover, the response to a high concentration of ligand to its cognate receptor led only to a two-fold stimulation relative to baseline for isoproterenol and to a more than 100-fold increase relative to its baseline for FSH. Although, a dose–response curve can be obtained for isoproterenol (Figure 5B), the experimental conditions will have to be improved in the future to reach a more satisfying response dynamic.

### 2.6. Kisspeptin Bioassays

**Figure 6:**
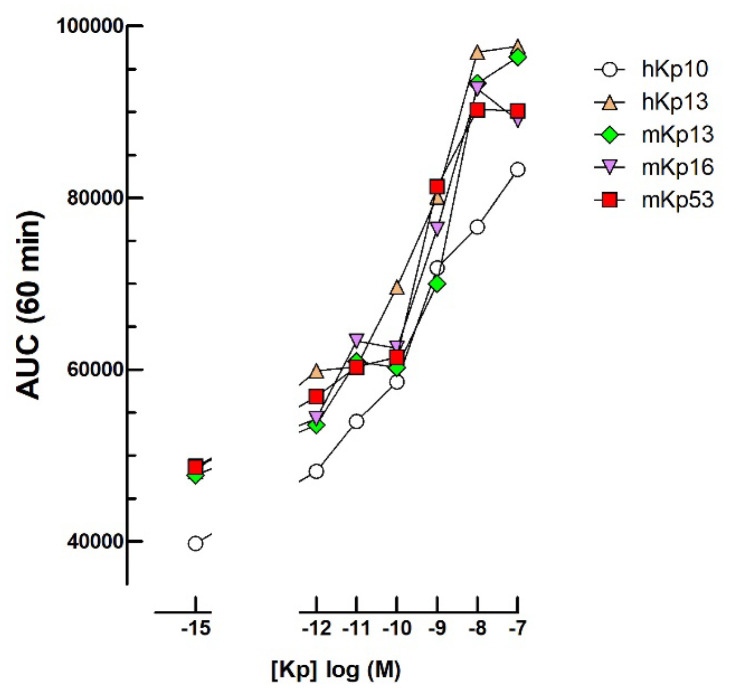
Dose-dependent cyclic AMP response to 10^−12^–10^−7^ M kisspeptin variants together with 0.3 nM oLH. The mLTC were transfected with 100 ng/well of the GPR54 hKp receptor plasmid before challenging with Kp and oLH added together at t48h for luminescence recording.

Following transfection of an expression vector for the human kisspeptin receptor (hKpR) at various concentrations, we observed, as expected, no cAMP response to 10^−12^–10^−7^ M Kp variants. However, we observed very similar dose-dependent synergies of all the tested Kp variants together with 3 × 10^−10^ M ovine LH (Figure 6).

### 2.7. Melatonin Bioassay

**Figure 7:**
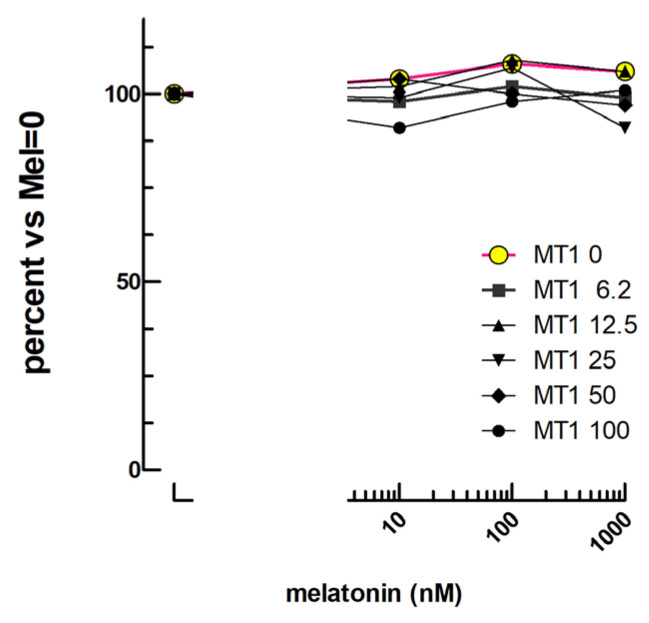
Absence of modulation of the cyclic AMP response to LH by melatonin. The mLTCs were transfected with various doses of the melatonin MT1 receptor expression vector (0 to 100 ng) and stimulated with 0.3 nM oLH in addition to the shown concentrations of melatonin.

Following transfection of the melatonin MT1 receptor (hMT1R) at various quantities (0 to 100 ng/well), we observed, as expected, no cAMP response to 10^−11^–10^−9^ M melatonin. But, moreover, we observed no negative effect on the cAMP response of mLTC to 3 × 10^−10^ M oLH with 10^−11^–10^−9^ M melatonin (Figure 7).

### 2.8. Oxytocin and Vasopressin Bioassays

**Figure 8:**
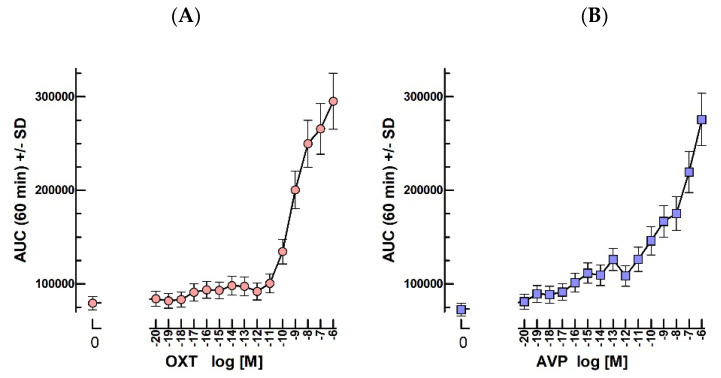
Effect of OT (**A**) or AVP (**B**) on the cyclic AMP response to hCG in mLTC. The mLTC cells were pre-incubated for 2 h with the shown concentrations of 10^−20^–10^−6^ M OT (**A**) or AVP (**B**) before stimulation with 2.5 × 10^−10^ M hCG and luminescence recording.

The pre-incubation with 10^−20^–10^−6^ M OT starting at the beginning of the 2 h-preincubation (t46h) led to a synergistic effect with 0.25 nM hCG introduced at the start of the luminescence recording for one hour (Figure 8).

There was a clear dose-dependent synergic effect of 10^−10^–10^−6^ M OT when hCG was added at t48h after the 2 h preincubation with OT (Figure 8A). As expected, in the same conditions, AVP exerted a similar effect over the same concentration range (Figure 8B).

## 3. Discussion

The expression of the GsPCR such as FSHR, TSHR, and PTHR allowed the setting up of in vitro bioassays in mLTC cells using the same protocol as for LH and hCG, which could be followed using the mLTCs’ endogenous LH receptors [1]. These in vitro bioassays were as sensitive as the previously described LH and CG assays. Although they can be used to evaluate the activities of the corresponding purified hormones, they are not specific, as contaminating LH or CG in serums, plasmas or other fluids would also be detected. Nevertheless, the overexpression of these different exogenous receptors, relative to the endogenous LH receptors, led to a diminished but still present response to LH (Figure 1B). An LHRKO mLTC cell line must be established for future transient GsPCR transfections to allow highly specific bioassays for their respective cognate ligands.

### 3.1. LH/CG

We previously reported [1] that all tested mammalian serums or plasmas could be assayed for their contents in bioactive LHs or CGs. In the present paper, we show that not only do serums or plasmas not negatively interfere but, in contrast, even promote a lower detection threshold of LH in the bioassay. It appears that this favorable effect on the detection limit of LH is due to the protein charge brought by sera during the serial dilutions of hormone before addition to the cells. Indeed, a similar effect was obtained when BSA was added at this step but not when added to the cells separately. It is a favorable point but, of course, requires that the hormone reference and sera are both prepared in BSA-containing medium to obtain valid dose–response curves. These threshold-lowering effects of serum or plasma have also been found for the FSH and TSH assays but have not yet been explored for the other hormones (PTH, Kp, ISO and MEL).

We have previously observed that chicken Luteinizing Hormone (ckLH) in sera could not be detected in our classical assay [1]. It will thus be necessary to transfect the ckLH receptor (ckLHR) in mLTC to hopefully obtain an in vitro bioassay for this hormone.

### 3.2. FSH

The expression of the hFSHR in the mLTC cells led to clear stimulation by hFSH at very low concentrations, below circulating concentrations in the blood. However, the response of the mLTCs to FSH was accompanied by a 50% drop in the response to LH. This decrease in the response to LH might be due to the lowering of LHR expression due to a competition with the transfected FSHR expression. It might also be due to LHR/FSHR hetero-dimerization despite their different species origins (mouse/human).

However, the FSH bioassay in its present state cannot specifically measure FSH concentrations in blood since LH still cross-reacts. It will be mandatory to delete the endogenously expressed LHR in mLTC (LHRKO mLTC cell line) to reach strict specificity in this highly sensitive FSH bioassay. The high responsiveness to FSH with this protocol suggests that it follows the same intracellular pathway, likely through the very early endosome (VEE) as the response to LH. In agreement with this, increasing concentrations of FSHR in the mLTCs lead to diminishing cAMP responses to LH (Figure 1B).

### 3.3. TSH

Similarly to the FSHR, the expression of the oTSHR in the mLTC cells led to clear stimulation by ovine TSH at very low concentrations, below circulating concentrations in the blood, thus allowing its specific detection in blood. We found very marginal cross-reaction by LH in the analyzed samples (Figure 2B) but this could also be due to the reproductive status of the ewes. The high response to TSH after TSHR expression in mLTC suggests that its downstream pathway is the same as that of LH probably through internalization of the TSHR to the VEE and subsequent stimulation of the soluble AdCy. Previous data have already demonstrated the importance of the soluble adenylate cyclase (AdCy10) in the TSH response pathway in another cell type [6]. As for the FSH bioassay, it will be required to use an LHRKO mLTC cell line to ascertain a high specificity of the TSH assay in mLTC.

The comparison of LHR, FSHR, and TSHR signaling pathways in mLTCs will be of high interest, as these receptors might compete but also potentially cooperate at different levels. For example, they can compete for GsPCR access but, also compete or cooperate by hetero-dimerization [7,8]. When both LHR and FSHR are co-expressed in HEK293 cells, an attenuation of the response to LH is observed, compared to LHR expression alone [7]. These data and our own (Figure 1), obtained in a different cell type with a different methodology, also points to attenuation of the LH response when the FSHR is overexpressed. This might be due to competition during the expression of the two receptors and/or to FSHR/TSHR hetero-dimerization.

The TSHR forms higher-order homomers rather than dimers [9,10]. It could thus be that such a higher oligomerization of TSHR might affect more strongly the LH response in mLTCs. Indeed, as the number of LHR in mLTCs is limited (3–15,000 per cell), it might be that the TSHR overexpression in these cells lower LHR expression or trap LHRs in TSHR/LHR hetero-oligomeric complexes, impeding LH stimulation.

### 3.4. PTH

The expression of the hPTHR in mLTC also allowed the detection of hPTH at very low concentrations, even lower than those of hFSH and oTSH in their respective bioassays in these cells. This better sensitivity of the PTH in vitro bioassay is well correlated with the known low Kd of PTH towards its receptor and its low circulating concentrations [11]. The present assay, by measuring PTH bioactivity, should permit to follow the activity of the injected PTH-Fc derivative at a very low concentration but could also exhibit extended half-life in the blood circulation [12].

### 3.5. Kisspeptins

The expression of the KpR in mLTC, led to a synergism by 10^−12^–10^−7^ M Kp variants with 3 × 10^−10^ M oLH. This synergy can be exploited for Kp bioactivity measurements but knowledge of the mechanisms involved would be necessary to ascertain specificity. This synergy might be due to an increase in cytoplasmic calcium concentration. Indeed, KpR through the Gq protein probably promotes stimulation of the phospholipase C/IP3 cascade leading to the liberation of endoplasmic reticulum- or mitochondria-sequestred calcium and, consequently, to further activation of AdCy10 activity [13,14]. Of course, it will be interesting to check this and other potential mechanisms to see whether active Kp in biological fluids can be specifically followed by this approach. This will certainly be difficult but this bioassay might be useful to design new drugs interacting with KpR.

### 3.6. β2AR Ligands

Upon transfection with the β2-adrenergic receptor (β2AR), we observed a weak cAMP accumulation in the absence of the agonist isoproterenol, and only a marginal additional stimulation in its presence. It can be hypothesized that either the β2AR was not correctly expressed in mLTC cells or did not interact efficiently with the cAMP signaling pathway in these cells. In mLTC, the FSHR, TSHR, and PTHR like the LHR largely act through the soluble AdCy pathway after internalization to the very early endosome [15]. This LHR internalization and intracellular stimulation appear dependent to the Gs protein, and thus one of the transmembrane adenylate cyclases (AdCy 1–9) in Nb37 cells [16,17]. It might be that the transmembrane AdCy specifically responsible for the isoproterenol β2AR stimulation in Nb37 and other cells is very weakly expressed in mLTCs, leading to its feeble activation. The β2AR was also shown to couple to Gi and to be unable to raise cAMP in specific cells such as mouse embryonic cardiomyocytes and canine ventricular myocytes. Perhaps, in mLTCs, the β2AR coupling to Gi is favored over Gs-coupling [18].

In addition, a recent report indicates that the C-terminal 71-aminoacid sequence β2AR (Ct) acts as an auto-inhibitory domain that interacts with the second and third intracellular receptor’s loops (ICL2 and ICL3), impeding Gs-protein interaction in the absence of agonist [19]. It could be that the weak intrinsic receptor activity and its low responsiveness to its agonist isoproterenol are correlated. If so, in the mLTC intracellular context, the Ct would establish an incomplete but persistent interaction with the β2AR ICL2 and ICL3. The only partial Ct interaction would explain the intrinsic receptor activity and its persistence would explain its low responsiveness in the presence of its agonist.

### 3.7. Melatonin

Similarly to the β2AR, the expression of the MT1 melatonin receptor in mLTC did not lead to any modulation of LH activity by its cognate ligand. In both cases, it can be due to insufficient expression of these exogenous receptors in mLTC or to a very unlikely deficient activity of the ligands. The expression of the two receptors led to a diminished response to LH similar to that observed after overexpression of FSHR, TSHR, or PTHR. It is thus unlikely that the β2AR and MT1 receptors were not expressed. Rather, they must activate downstream pathways different from those of LHR, FSHR, TSHR, or PTHR. Indeed, we have previously observed [15] that the LH-stimulated cAMP accumulation in mLTC cells might essentially be due to the type 10 adenylate cyclase (AdCy10), also known as soluble adenylate cyclase (sAC) which is insensitive to G-protein modulation but has been found responsive to Ca_2+_, pH, and ATP [16]. The MT1R, which is known to act through the Gi protein [20], would thus be inactive on the AdCy10 activity stimulated by LH in mLTC, thus explaining that melatonin did not inhibit LH activity in these cells.

### 3.8. OT

The present bioassay has been set up without transfecting exogenous OTR and making use only of the mLTCs’ endogenous receptors. It permits to assay oxytocin by introducing it during the preincubation period before adding a fixed dose of LH or hCG. AVP, which is known to partly act through the OTR, was also found to exert a similar dose-dependent effect on the hCG response in the bioassay.

We have previously shown that the OT and AVP effects are dose- and time-dependent and persist when LH or hCG is added after their removal [1]. This demonstrates that these hormones potentiate LH/CG activities through the intracellular accumulation of an intermediate of the endpoint cell response. Potentially, this intermediate might be the recombinant cAMP-dependent luciferase itself as oxytocin is known to stimulate protein translation in many cell types [21,22,23].

## 4. Materials and Methods

### 4.1. Protocols

The assays presented in this paper are based on our previously described protocol for luteinizing hormone (LH) and chorionic gonadotropin (CG) in vitro bioassays [1]. The mouse Leydig Tumor Cells (mLTC) were obtained from the American Tissue and Cell Collection (ATCC) (LGC Standards, Molsheim, France) and have been regularly tested negative for mycoplasma contamination all over our studies. As previously reported [24], the mLTCs, between their 6th and 30th passage, were used.

At t0, the cells were trypsinized, counted, transfected and transferred (~80,000 cells per well) to 96-well Greiner white/clear-bottom plates (Dutscher, Bernolsheim, France). They were then expanded at 37 °C under 5% CO_2_ in 200 μL RPMI-1640 growth medium (Gibco, Invitrogen, Waltham, MA, USA), supplemented with 10% fetal calf serum (FCS), 50 µg/mL gentamicin, 10 units of penicillin/mL and 10 µg/mL streptomycin

At t24h, the cell culture medium was removed to eliminate the transfection reagents and replaced with RPMI medium containing FCS and antibiotics as above.

At t46h, 1.0 mM 1-Methyl-3-Isobutylxanthine (IBMX) was introduced in all experiments. It inhibits the nucleotide phosphodiesterase (PDE) activity in mLTCs to avoid the degradation of 3′5′cAMP into 5′AMP and thus amplify its accumulation. Forskolin (FSK), oxytocin (OT) and 1 µL per well of the Glosensor substrate (Promega France, Charbonnières, France) were also added at the beginning of the 2 h preincubation. In contrast to most other cell lines, 10 µM forskolin (FSK) alone in mLTCs exerts no stimulation of cAMP accumulation [24] but had a strong synergy with LHs and CGs in eliciting the cAMP response of mLTCs [1,24]. Moreover, to increase the amplitude of the cAMP response of mLTC, 10^−8^ M OT was added, from the beginning of the 2 h preincubation period on [1]. Concerning OT and AVP assays, these two hormones were added at various concentrations at t46h before the addition at t48h of a fixed concentration of LH or hCG during the luminescence measurement.

**Figure 9:**
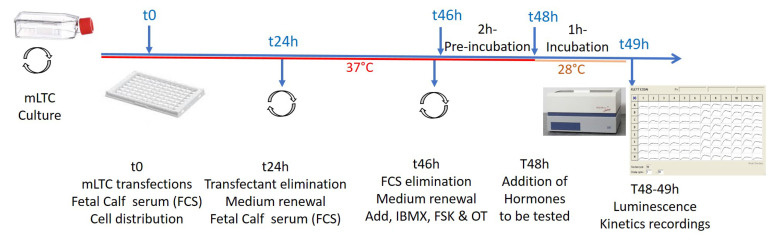
Scheme of protocols’ steps. One plate 96 parallel real-time luminescence kinetics recordings (usually 32 kinetics in triplicates for each condition). For details, see text.

Transfections of cells with 100 ng/well pGlosensorTM 22F cAMP plasmid (Promega France, Charbonnières, France) were performed using 0.3 µL/well XtremeGENE HP DNA transfection reagent (Roche Diagnostic France, Meylan, France), allowing the expression of a cAMP-dependent luciferase. When required, 1 to 100 ng exogenous receptor expression vectors (FSHR, TSHR, PTHR, KISS1R, βAR or MT1R) were co-transfected with the Glosensor luciferase plasmid at t0, 48 h before the mLTC stimulation by the corresponding hormone under study. Hormone serial dilutions were carried out in PCR 96-tube plates (Thermo-scientific #AB-0900, Waltham, MA, USA) just before t48h (Figure 9), generally in RPMI medium alone. In some experiments, 1 mg/mL bovine serum albumin (BSA, Sigma-Aldrich #A7906, St. Louis, MO, USA) was present at this stage.

For the sake of clarity, the scheme in Figure 9 recapitulates the protocol’s steps.

### 4.2. Hormones

Human Choriogonadotropin hCG (7482 UI/mg) was from Hepartex (Saint-Cloud, France), recombinant human LH (4500 IU/mg) from Sigma-Aldrich (Merck, Darmstadt, Germany), a pituitary hLH (SIAFP-4261A)), and highly purified equine LH (AFP-50130A), ovine TSH (AFP-3748B) and human FSH (hFSH SIAFP1) from NHPP (Torrance, CA, USA). Ovine LH (oLH CY1083; 3.0 × NIH LHS1) and bovine LH (bLH CY1280; 1.2 NIH LH B1) were purified in our laboratory by adapting the previously described procedure by Hennen et al. for porcine LH [25]. The various KISS1 isoforms were obtained from Tocris Bioscience (Bristol, UK). Melatonin (MLT) and Isoproterenol hydrochloride (ISO) were from Merck (Darmstadt, Germany). Oxytocin (OT), Arginine-vasopressin (AVP) and human Parathyroid Hormone (PTH) 1–34 were obtained from Bachem AG (Budendorf, Germany).

### 4.3. Receptors’ Expression Vectors

#### 4.3.1. Human FSH Receptor

The plasmid encoding the full-length human FSHR–FLAG fusion protein was inserted in the pcDNA3.1+ vector (GenScript, Rijswijk, The Netherlands) employing the procedure previously described to construct the rat FSHR–FLAG [26]. The original full-length human FSHR cDNA was obtained from the Missouri S&T cDNA Resource Center (Rolla, MO, USA). 

#### 4.3.2. Ovine TSH Receptor

The expression vectors for ovine TSHR (GenBank accession number NM_001009410) and the ovine MTNR1A (GenBank accession number NM_001009725) encode for fusion TSHR and MTNR1A receptor sequences harboring a 5X-Myc tag on their N-terminus and have been thoroughly validated previously using COS7 cells [27,28].

#### 4.3.3. Human PTH Receptor

The plasmid encoding for the HA-hPTH1R [29] (a gift from Pr. Jean-Pierre Vilardaga, University of Pittsburgh, Pittsburgh, PA, USA) was subcloned in the pcDNA3.1+ vector for expression in mLTCs. 

#### 4.3.4. Human KISS Receptor

The human KISS1R (GPR54; GenBank accession number NM_032551; with SNP rs350132) was cloned in the same vector backbone from a HEK293 cell line stably expressing the receptor and used for screening purposes [30,31]. The coding sequence was inserted in the expression vector pcDNA3.1+ (GenScript, Rijswijk, The Netherlands) for expression in mLTCs.

#### 4.3.5. Human β2-Adrenergic Receptor

The pcDNA3 Flag beta-2-adrenergic-receptor expression vector (Addgene plasmid # 14697) was a gift from Pr. Robert Lefkowitz (Duke University, Durham, NC, USA).

### 4.4. Biological Samples

For the TSH assays in mLTC, ovine blood samples were taken from intact and thyroidectomized ewes (i.e., just before and after surgery) and had been previously assayed for TSH using a specific RIA [32].

### 4.5. Area Under Curve (AUC) Calculations and Statistical Analyses

The GraphPad 5.0 (GraphPad Software, San Diego CA, USA) was used for Area Under Curve (AUC) determinations of individual kinetics. Mean and SD values for each triplicate AUCs were determined. The AUC ratios were used to compare cAMP response kinetics. One-way ANOVA with Dunnett’s test was also performed using this package. The level of significance was *p* < 0.05.

## 5. Conclusions

Overall, the data reported here show that it is feasible to set up highly sensitive in vitro bioassays for hormones other than LH or CG using the mLTC cell line. For those acting through Gs protein and probably soluble adenylate cyclase (like FSH, TSH, PTH), the kinetics of intracellular dose-dependent cAMP accumulation followed by luminescence, allow us to directly evaluate the bioactivities of samples. For the other hormones and neuromediators like OT, AVP, or Kp, their modulations of the cAMP response to a fixed dose of LH/hCG, can be used as endpoints.

## Data Availability

Data are contained within the article.
